# TECHNOLOGICALLY MEDIATED INTERVENTIONS IN HOSPICE AND PALLIATIVE CARE: CONSIDERATIONS FOR RURAL ELDERS

**DOI:** 10.1093/geroni/igz038.720

**Published:** 2019-11-08

**Authors:** Karla T Washington

**Affiliations:** University of Missouri, Columbia, Missouri, United States

## Abstract

Rural communities are home to a disproportionate number of older adults, many of whom are living with a serious illness or providing care to a seriously ill family member or friend. For these individuals, hospice and palliative care services can provide much-needed biopsychosocial and spiritual support, leading to an enhanced quality of life. Technologically-mediated interventions hold promise as a strategy to bridge geographic distances between healthcare providers and rural elders; however, the effect of such interventions may be greater when they are designed in a manner congruent with rural culture and compatible with the technological infrastructure common in rural areas. Informed by lessons learned during a pilot study of a telehealth intervention in one Midwestern state (R21CA191165), this presentation includes a discussion of strategies to more effectively recruit, engage, and retain rural older adults into studies testing technologically-mediated interventions for hospice and palliative care patients and their families.

